# Development and Optimization of a Highly Sensitive Sensor to Quinine-Based Saltiness Enhancement Effect

**DOI:** 10.3390/s23063178

**Published:** 2023-03-16

**Authors:** Yifei Jing, Kentaro Watanabe, Tatsukichi Watanabe, Shunsuke Kimura, Kiyoshi Toko

**Affiliations:** 1 Graduate School of Information Science and Electrical Engineering, Kyushu University, 744 Motooka, Nishi-ku, Fukuoka 819-0395, Japan; 2 Research and Development Center for Five-Sense Devices, Kyushu University, 744 Motooka, Nishi-ku, Fukuoka 819-0395, Japan; 3 Institute for Advanced Study, Kyushu University, 744 Motooka, Nishi-ku, Fukuoka 819-0395, Japan

**Keywords:** taste sensor, lipid/polymer membrane, saltiness enhancement effect, saltiness sensor, ionophore

## Abstract

The saltiness enhancement effect can be produced by adding specific substances to dietary salt (sodium chloride). This effect has been used in salt-reduced food to help people forge healthy eating habits. Therefore, it is necessary to objectively evaluate the saltiness of food based on this effect. In a previous study, sensor electrodes based on lipid/polymer membrane with Na^+^ ionophore have been proposed to quantify the saltiness enhanced by branched-chain amino acids (BCAAs), citric acid, and tartaric acid. In this study, we developed a new saltiness sensor with the lipid/polymer membrane to quantify the saltiness enhancement effect of quinine by replacing a lipid that caused an unexpected initial drop in the previous study with another new lipid. As a result, the concentrations of lipid and ionophore were optimized to produce an expected response. Logarithmic responses have been found on both NaCl samples and quinine-added NaCl samples. The findings indicate the usage of lipid/polymer membranes on novel taste sensors to evaluate the saltiness enhancement effect accurately.

## 1. Introduction

Taste evaluation has been used in the development as well as the quality management of foods, beverages, and pharmaceuticals. Commonly, the sensory test and the chemical analysis are methods of quantifying the tastes of the products. However, the sensory test is affected by the physical and psychological conditions of testers, making the results subjective. The chemical analysis provides data on each substance in the food, but it can neither demonstrate the overall taste nor the taste-substance interactions.

Under such conditions, sensors that can quantify or identify taste have been developed to solve the problems [1,2,3,4,5,6]. The electronic tongues (e-tongues) are nonspecific sensor arrays dedicated to the analysis of complicated liquids such as wine, and juice [2,7,8,9]. They are capable of distinguishing similar liquids because the sensors used in the sensor array can be selected to be very different to make the sensor able to measure a broad range of substances in the solution. The electronic tongues have been widely used in the quality control of wine, juices, and beverages [2,3,4,5,6,8]. The data produced by the array is processed by recognition algorithms so that the result is readable by human. Data analysis methods such as principal component analysis (PCA), machine learning, and artificial neural networks have been used to process the multi-dimensional data from the sensor array [2,3,4,5,6,7,8,9].

The taste sensor based on lipid/polymer membrane has been developed with the concept of global selectivity, which states for the selectivity to all chemical substances of individual taste so that the taste sensor is able to perceive the taste of any product [10,11,12,13]. It is proposed under the idea of imitating the mechanism of the human gustatory system which is capable of sensing each taste rather than a specific taste substance. The taste sensor is capable of quantifying the intensity of particular taste quality based on the responses of the sensor electrodes which are potential changes of lipid/polymer membranes [10,11,12,13,14], and the quantities can be linearly converted into human scale because the relationship between the response and concentration of the sample is consistent to the Weber-Fenchner law, which states that the perceived change of human is proportional to the logarithmic of physical change [10,13].

The main difference between the e-tongues and the taste sensors is that the result from the e-tongues demonstrates the information to differentiate types or states of food (such as beers and oils), while the result from the taste sensors is a quantity to show the intensity of the taste.

The lipid/polymer membranes, which are made of lipids, plasticizers, and polyvinyl chloride (PVC), interact with the taste substances mainly based on electrical and hydrophobic properties. The electrical property originates from lipids because they dissociate in the membrane to contain charges. The hydrophobicity is from all of the components because they all contain hydrophobic groups. It is possible to quantify one taste quality selectively by changing the type and amount of lipids and plasticizers. For instance, sensor of saltiness taste CT0 contains a lipid called tetradodecylammonium bromide (TDAB) which is positively charged to make the sensor sensitive to chloride ions of NaCl, and a plasticizer called dioctyl phenylphosphonate (DOPP) which makes the membrane hydrophilic so that the sensor is not responsive to bitter substances which are hydrophobic.

The taste sensor has been utilized in food and beverage manufacturing to control the taste of the food [15,16,17] and give out quantified food information to consumers [18]. In the pharmaceutical field, the taste sensing system helps to optimize the ingredients of the medicine by alleviating the bitterness taste, which is called bitterness masking [19,20,21,22]. However, since the taste sensor is based on electro-chemical environment of the sample solution, it is not possible to measure other organoleptic characters such as aroma and texture feel. Furthermore, as the perception of taste is also dependent on the odor [23,24], the response is sometimes subjective. Re-usability problems have also been found on the bitterness sensors composing phosphoric acid di-n-decyl ester (PADE) [25].

The tastes perceived by humans are defined as five basic tastes: saltiness, sourness, sweetness, bitterness, and umami (savoriness) [26]. Each of these tastes has biological features and specific information. For example, saltiness is mainly caused by metallic materials like sodium ions. It brings a signal of minerals to the body and indicates the electrolyte balance of the food. Dietary sodium has been commonly used to increase the deliciousness of food in daily life. However, excessive intake of salt has been proven to be associated with some diseases, such as hypertension [27]. A report from the World Health Organization states that people consume 9–12 g of salt on average per day, which is almost twice adults’ recommended intake (5 g salt per day) [28]. Therefore, it is required to decrease the amount of salt in food while keeping the salty taste rarely changed.

The saltiness enhancement effect is the effect to intensify the salty taste by adding specific substances to salt (sodium chloride). Such substances, called saltiness enhancement substances, usually have other taste qualities even when the saltiness enhancement effect do not occur: umami taste substances such as monosodium glutamate (MSG), branched-chain amino acids BCAAs; sour taste substance such as tartaric acid and citric acid [29,30,31,32], and bitterness taste substance such as quinine hydrochloride dihydrate. However, neither the structures of such substances nor their varieties demonstrate a pattern to explain why the saltiness enhancement effect should occur; it is still unclear the exact mechanism of the saltiness enhancement effect, and the development of salt-reduced food is dependent on sensory tests.

Work has been done on the development of a taste sensor on saltiness enhancement effect [33]. This taste sensor can measure the saltiness enhancement effect produced by BCAAs, citric acid, and tartaric acid, using an ionophore called bis[(12-crown-4)methyl] 2-dodecyl-2-methylmalonate and a lipid called PADE. Ionophores are generally hydrophobic compounds that act as ion-exchanger sites on the ion-selective electrode (ISE) [34]. Bis[(12-crown-4)methyl] 2-dodecyl-2-methylmalonate is an ionophore that is highly selective to sodium ions because the diameter of the two crown rings can fit perfectly for a sodium ion [35]. PADE is a lipid that is negatively charged in the membrane and has been used in the bitterness sensors [14,25,36]. With the hydrophobicity and negatively charging property, the sensor should be able to detect the saltiness enhancement substances. However, the sensor responses did not increase monotonically with these substances at low PADE concentrations, introducing an initial drop. This is because of the partial dissociation property of PADE [33]. Further investigation on the dissociation property of PADE and tetrakis[3,5-bis(trifluoromethyl)phenyl]borate sodium salt dehydrate (TFPB) has been done in [37], where membranes made from these two substances have shown differences on ion selectivity; membranes made from PADE had low ion selectivity while high ion selectivity had been found in TFPB membranes.

In this study, a new lipid/polymer membrane was fabricated without the use of partially dissociated PADE to get rid of the initial potential drop problem. This sensor was expected to measure the saltiness enhancement effect produced by quinine, which contains a positively charged intramolecule [38] and hydrophobic groups. The substance selected to replace PADE is TFPB because it is fully dissociated to solve the initial drop problem and it is also negatively charged in the membrane to act as counter cites to quinine. To increase sensitivity, the amount of ionophore in the sensor was optimized.

As a result, the taste sensor using the membrane comprising TFPB and Na^+^ ionophore satisfied the requirements for measuring dietary salt sample and the quinine added saltiness enhancement sample.

## 2. Materials and Methods

### 2.1. Sensory Test

We performed a sensory test on the saltiness enhancement effect in collaboration with the Taste & Aroma Strategic Research Institute Co., Ltd. (Tokyo, Japan) in a similar way [33]. Ten panelists took the two-point identification test. After cleaning their mouth with pure water, they should evaluate two samples without being told the compositions and give out which is saltier or almost the same. We tasted two types of solutions of NaCl solution and NaCl+quinine solution, whose compositions are two and three different concentrations of NaCl (0.375%, 1%) and quinine (0.0027 mM, 0.008 mM, 0.024 mM), respectively. The quinine concentration was chosen around its human threshold, as found in most saltiness enhancement substances, because it is several µM [39,40,41].

### 2.2. Reagents

**Figure 1:**
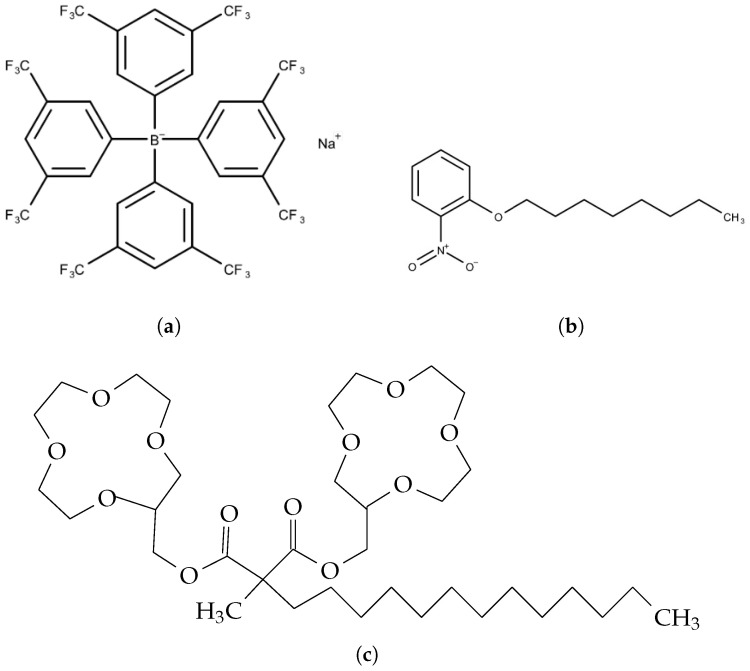
Structure of reagents: (**a**) TFPB. (**b**) NPOE. (**c**) Bis[(12-crown-4)methyl] 2-dodecyl-2-methylmalonate (Na^+^ ionophore).

For the components of membranes, sodium ion selective ionophore bis[(12-crown-4)methyl] 2-dodecyl-2-methylmalonate (Na^+^ ionophore), TFPB, and polyvinyl chloride (PVC) were purchased from FUJIFILM Wako Pure Chemical Corporation (Osaka, Japan), 2-nitrophenyl octyl ether (NPOE) and tetrahy-drofuran (THF) were purchased from Sigma-Aldrich Japan G.K. (Tokyo, Japan). The structures of these substances are shown in Figure 1.

### 2.3. Lipid/Polymer Membrane

In this study, to investigate the effects of the Na^+^ ionophore on the electrical properties of membranes, lipid/polymer membranes composed of different concentrations of Na^+^ ionophore were fabricated. The details of the membranes are shown in Table 1.

For the samples, quinine hydrochloride dihydrate, tartaric acid, potassium chloride (KCl), and sodium chloride (NaCl) were purchased from Kanto Chemical Co., Inc. (Tokyo Japan).

First, Na^+^ ionophore (5 g) was dissolved in THF (5 mL). Later the calculated amount of Na^+^ ionophore solution was mixed into this vial snap. The lipid/polymer membranes were fabricated under the procedures as shown below:

1. Prepare a 45 mm petri dish and a 20 mL vial snap;
2. Dissolve TFPB, NPOE, and PVC with 10 mL THF in the vial snap;
3. Add Na^+^ solution ionophore accordingly into the vial snap.
4. Stir the vial snap for 1 h and pour the content into the petri dish.
5. Dry the petri dish for three days in a draft chamber with a temperature of 25 °C to let the THF be fully volatilized.

A well prepared membrane was a transparent and colorless film about 300 μm thick.

### 2.4. Samples

To test the response of the sensors, two kinds of samples were prepared: NaCl solution and NaCl + quinine solution. The NaCl solution was to test whether the sensor could respond to normal saltiness. The NaCl + quinine solution was to test whether the sensor could respond to the saltiness enhancement effect caused by quinine. The solutions were all made with deionized water. The details of the samples are shown in Table 2.

Additionally, a standard solution, made of 30 mM KCl and 0.3 mM tartaric acid, was prepared to initialize the sensor; a cleaning solution, made of 100 mL HCl and 30 vol% ethanol, was prepared to wash the samples left on the surface of the sensor after each measurement [42].

### 2.5. Sensor Preparation

For the preparation of working electrodes, the membranes made in Section 2.3 were cut and pasted onto the sensor probe. An adhesive (a mixture of 10 mL THF and 800 mg PVC) was used to paste the membranes. The probes were then filled with a solution of 3.33 M KCl and saturated AgCl. After that, an Ag/AgCl electrode was inserted into the probe and the probe should be immersed into the standard solution for at least 72 h to complete the process of pre-conditioning, which is to align the lipids on the membrane surface so that the hydrophilic parts of the lipids facing toward outside [10,13,14,43,44].

Sensor electrodes were then connected to a unit of TS-5000Z Taste Sensing System, which is a commercialized taste sensing system from Intelligent Sensor Technology, Inc., Kanagawa, Japan. Each unit can connect to 4 working electrodes and 1 reference electrode [10,14,33].

The construction of this measuring system is as follows: Ag wire coated with AgCl|3.33 M KCl and sat. AgCl solution|membrane|sample solution|reference electrode (Ag/AgCl) in taste solution. Changes in potentials were measured and recorded in the taste sensing system.

### 2.6. Measurement Procedures

The measurement procedures had four steps as stated in [10,14]. First, the sensor was immersed in the standard solution for 30 s to measure the reference potential (V_r_). Then the sensor was immersed in the sample solution for 30 s to measure the sample potential (V_s_). After that, the sensor was again immersed in the standard solution to obtain an adsorbed reference potential (V^${}^{\prime }$^_r_). Finally, the sensor was immersed in the cleaning solution to remove adsorbed substances on the membrane surface. The use of TS-5000Z Taste Sensing System is to automatize the measurement procedures [10,14]. The difference of V_s_ and V_r_ is called the relative value, or response value, which is to quantify the initial taste. The difference of V^${}^{\prime }$^_r_ and V_r_ is called CPA (Change of membrane Potential caused by Adsorption), which is to measure the taste produced by substances adsorbed on the membrane [10,14]. These substances are usually bitter and astringent substances, which are adsorbed strongly on the human tongue. The CPA value corresponds to the aftertaste perceived by humans and depends on the state of the charge of the membrane as well as the amount of taste substances adsorbed onto the membrane [45].

The steps mentioned above compose a cycle, and 5 cycles are performed in one measurement. The average and standard deviation of the second to fifth cycle was calculated as the response value [14].

## 3. Results

### 3.1. Saltiness Enhancement Effect of Quinine

The saltiness enhancement effect of quinine was confirmed from the sensory test. The sensory test on the saltiness enhancement effect was performed in collaboration with the Taste & Aroma Strategic Research Institute Co., Ltd. (Tokyo, Japan). Ten well trained panelists (four male and six female adults) were enrolled in this study. The panelists were selected by their ability to detect taste substances with high sensitivity, and have been trained approximately once a week. The result is showed in Table 3. It can be found that when the concentration of NaCl was low (0.375%), the quinine did have the saltiness enhancement effect on NaCl. When the concentration of NaCl was relatively high (1%), the quinine did not have the saltiness enhancement effect.

From this result, the taste sensor on saltiness enhancement effect should produce a increasing response when quinine is added to low concentration NaCl solution, while staying unchanged when quinine is added to high concentration NaCl solution.

### 3.2. Response to NaCl

**Figure 2:**
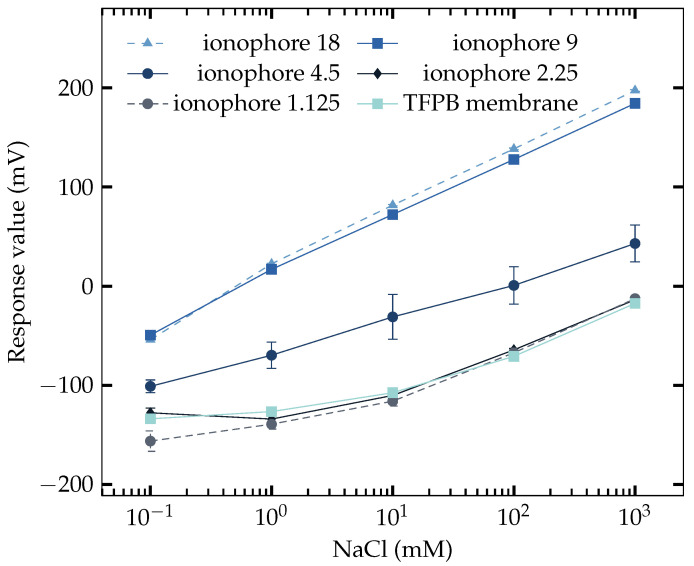
Responses of TFPB membrane and NaISE to NaCl solutions. The responses of NaISE are name after the amount of Na^+^ ionophores used.

Because it is needed to satisfy the basic function as a saltiness sensor and test the effect of the Na^+^ ionophore, the responses to NaCl samples were tested on two types of membranes with the compositions listed in Table 4. The results are shown in Figure 2, where both of the membranes behaved monotonically increasing with the NaCl concentration, while the response of Na^+^ ion-selective electrode (NaISE) is much better. The results were separated into two groups: the ones over 4.5 mg and the ones below 4.5 mg showed almost the same response respectively. The response of TFPB membrane is the same as reported [37]. This result has shown that the Na^+^ ionophore can dramatically improve the sensitivity of NaCl.

### 3.3. Response to Saltiness Enhancement Samples

**Figure 3:**
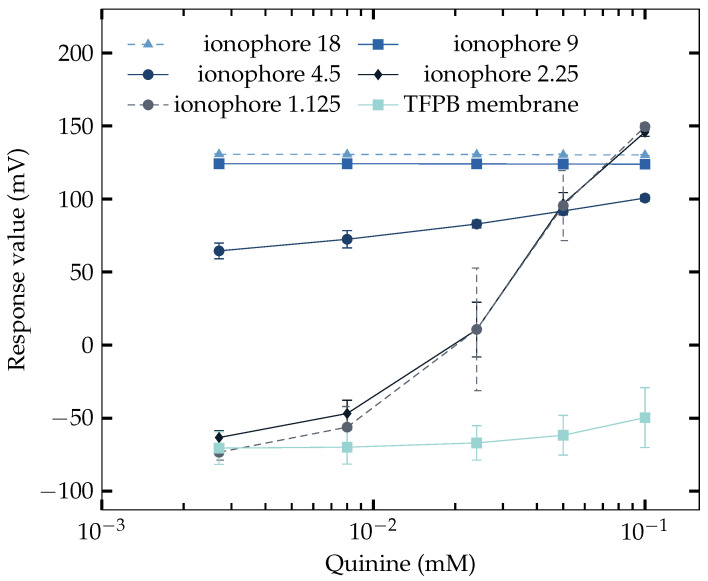
Responses of NaISE of different Na^+^ ionophore concentrations to NaCl (70 mM) + quinine solutions.

The same type of membranes (in Table 4) were used in the measurement of saltiness enhancement samples (NaCl + quinine). The concentration of NaCl was 70 mM as it is the concentration when the saltiness enhancement effect is produced in Table 3; hence increasing response should be generated by the sensors. The result is shown in Figure 3, where some of the membranes failed to determine the saltiness enhancement effect. It can be found that when the amount of Na^+^ ionophore was 4.5 mg to 1.125 mg, good sensitivity of the saltiness enhancement effect can be achieved. It is intriguing that the results are not clearly divided into two groups as in Section 3.2, while neither the ones with high amount of Na^+^ ionophore nor the ones only with TFPB were able to measure the saltiness enhancement effect. Only the ones with Na^+^ ionophore lower than 4.5 mg showed the saltiness enhancement effect. The response of TFPB membrane started to increase from about 0.02 mM. However, as it is required to measure the saltiness enhancement effect with the concentration of quinine in Table 3, the TFPB membrane is required to reach more sensitivity before 0.008 mM of quinine.

### 3.4. Saltiness Enhancement Effect Measurement

**Figure 4:**
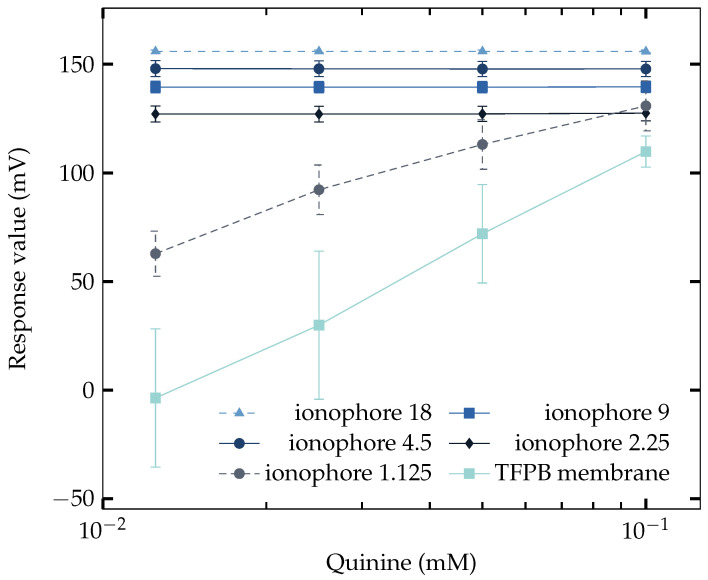
Responses of NaISE of different Na^+^ ionophore concentrations to NaCl (200 mM) + quinine solutions.

To make sure that the saltiness enhancement effect of quinine can only be detected at the relatively low concentration of NaCl, the measurement under high level concentration of NaCl (200 mM) has also been performed. The results are shown in Figure 4, where the NaISE with 1.125 mg Na^+^ ionophore added and the TFPB membrane had large sensitivity to quinine. The result, on the other hand, showed that the concentration of NaCl in the solution can affect the sensitivity to quinine. We can conclude that the taste sensor composed of Na^+^ ionophore and TFPB can detect the saltiness enhancement effect at lower NaCl concentrations by comparing the two results in Figure 3 and Figure 4, which agrees with human sensory evaluation shown in Table 3.

### 3.5. Adsorption Property

**Figure 5:**
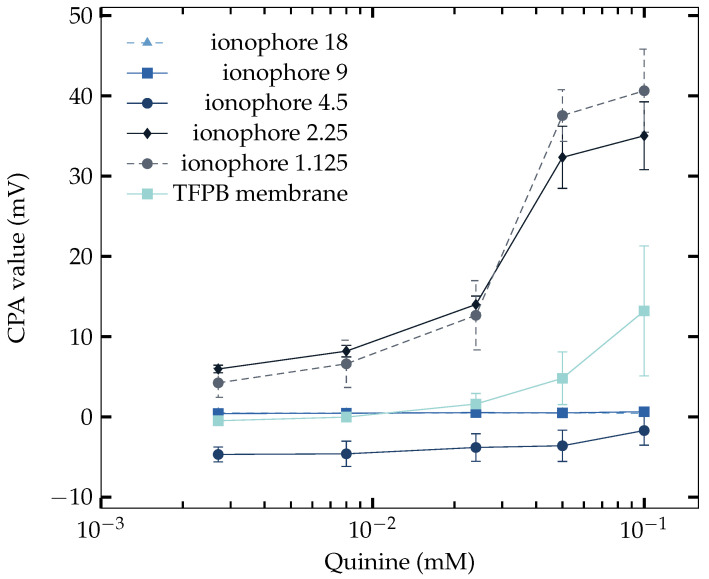
CPA responses of NaISE of different Na^+^ ionophore concentrations to NaCl (70 mM) + quinine solutions.

To explain why only the membranes with 2.25 and 1.125 mg Na^+^ ionophore added succeed under 70 mM NaCl, the CPA value, which demonstrates the adsorption property of the membrane, is plotted in Figure 5, where only the membranes with 2.25 and 1.125 mg Na^+^ ionophore added appeared to adsorb quinine on the membrane surface. This fact is considered to be a strong evidence that the increasing response is resulted from quinine.

### 3.6. Proposal Composition of NaISE

**Figure 6:**
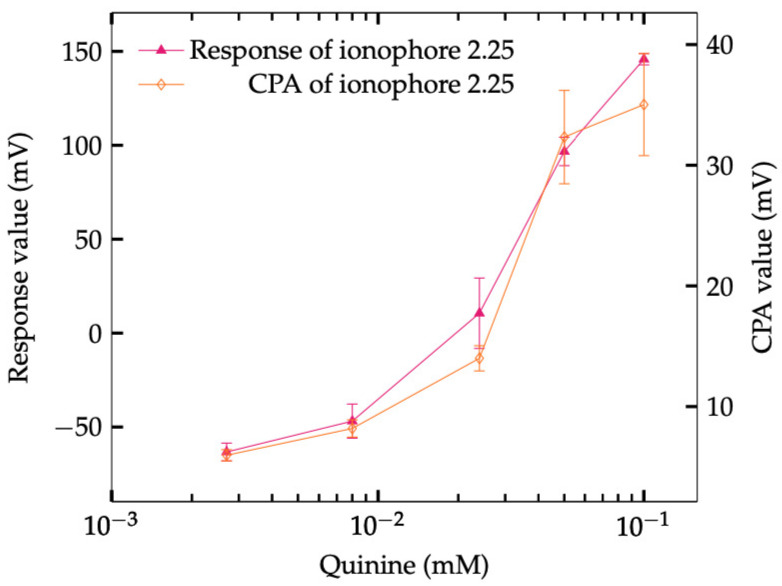
CPA Responses of NaISE of different Na^+^ ionophore concentrations to Quinine + NaCl (70 mM) solutions.

From the results in previous sections, it is reasonable to conclude that the best amount of the Na^+^ ionophore is 2.25 mg, as it can determine the saltiness enhancement effect at the right concentration of NaCl. The response value and the CPA value of this membrane are shown in Figure 6, where the two properties of the membrane had depicted large correlation.

## 4. Discussion

In Figure 3 using 70 mM NaCl, it can be found that the sensor started to be able to measure the saltiness enhancement effect when the amount of Na^+^ ionophore reached a low level (below 4.5 mg). The sensitivity increased as the amount of ionophore became lower. Without the use of Na^+^ ionophore, the sensor again failed to measure the effect. Therefore, it is the ionophore that handle the character of measuring quinine in the NaCl solution.

A similar phenomenon has also been found in PVC and DOPP membrane [46], where the response to quinine decreased when the concentration of lipid increased, implying that the magnitude of hydrophobicity decreased due to the hydrophilic charged group of lipid. However, it also provides evidence that a membrane contains PVC and plasticizer can have great hydrophobicity and is able to act as a negatively charged membrane to be sensitive to quinine. If it is the PVC and plasticizer that are responsible to the measurement of quinine, the decrease of ionophore in this experiment is actually a process of changing the ratio of them in the membrane. The result in Section 3.4 using 200 mM NaCl showed that the hydrophobicity can also be affected by the concentration of NaCl. The reason that the sensors with a high amount of ionophore failed on sensing the saltiness enhancement effect is understandable because of the screening effect created by sodium ions on the surface of the membrane. The electrical double layer can behave positively charged, with Na^+^ ions accumulating on the surface of the membrane, thus repelling the quinine. The evidence can be found while finding the response of the sensor under a concentration of 200 mM of NaCl solution. As long as the concentration of Na^+^ ions increased in the solution, the amount of Na^+^ ions is greater, and the screening effect should be stronger. Thus, the saltiness enhancement effect appears at lower ionophore concentrations (from ionophore 1.125, not ionophore 2.25) in Figure 4. Another piece of evidence is that the response and CPA of ionophore 2.25 mg showed a high correlation in Figure 6. This implied that quinine has been adsorbed on the membrane surface. It can also be found in Figure 5, where the occurrence of CPA showed up at the same time as being able to measure the saltiness enhancement effect.

The TFPB membrane scarcely responded to quinine at coexistent 70 mM NaCl in Figure 3, whereas it largely responded to quinine at coexistent 200 mM NaCl in Figure 4. This difference is because the adsorption effect of quinine on the membrane potential is large when the TFPB membrane becomes electrically neutralized by high NaCl concentrations, as indicated in [45].

## 5. Conclusions

In this study, we showed that the saltiness enhancement effect can be produced from quinine when the concentration of NaCl was low, and we successfully developed a sensor to quantify the saltiness enhancement effect produced by quinine hydrochloride dihydrate. The sensor was designed to get rid of the use of the lipid PADE, owing to the initial drop problem. To increase the sensitivity and selectivity, the amount of Na^+^ ionophore was modulated, so that the taste sensor can not only measure the saltiness enhancement effect correctly (when the effect is generated under low salt concentration) but also sustains a large response region. The reason why the sensor was able to measure the effect only when the ionophore concentration was low is that the hydrophobicity of the membrane changed because of the ratio of ionophore to PVC and plasticizer is adjusted. The reason that the sensor failed to measure the effect under a high amount of Na^+^ ionophore has been discussed (decreased hydrophobicity and increased screening effect). The results from trials showed that the composition of 2.25 mg Na^+^ ionophore in the membrane is very suitable for the measurement of the saltiness enhancement effect produced by quinine. Because the mechanism of the saltiness enhancement effect has not been established, it is only possible to design a sensor which can imitate the saltiness enhancement effect obtained from sensory test. Currently, the salt-reduced foods using the saltiness enhancement effect produced from quinine have not been developed yet. The development of this sensor is assumed to be supportive to novel salt-reduced food products base on quinine. Future work can be done on investigating whether this sensor can measure the saltiness enhancement effect produced by commercialized substances.

## Figures and Tables

**Table 1:** Components and concentrations of membranes.

Component	Amount
Na^+^ ionophore	18, 9, 4.5, 2.25, 1.125 mg
TFPB	1.5 mg
NPOE	400 uL
PVC	200 mg

**Table 2:** Components and concentrations of samples.

Sample	Concentration
NaCl	0.1, 1, 10, 100, 1000 mM
NaCl + quinine	NaCl (70, 200 mM) + quinine hydrochloride dihydrate (0.0027, 0.008, 0.024, 0.05, 0.1 mM)

**Table 3:** Results of sensory test on quinine. The data is the number of panelists who decided which of the two samples was saltier. The comparing samples are NaCl solutions and the same NaCl solutions with quinine added.

Comparaing Samples		NaCl + Quinine	NaCl	Same
NaCl (0.375%)	+ quinine (0.0027 mM)	10	0	0
	+ quinine (0.0080 mM)	10	0	0
	+ quinine (0.024 mM)	10	0	0
NaCl (1%)	+ quinine (0.0027 mM)	0	10	0
	+ quinine (0.0080 mM)	0	10	0
	+ quinine (0.024 mM)	1	9	0

**Table 4:** The composition of the TFPB membrane and the NaISE.

Membrane	Plasticizer & Polymer Support	Additional Compositions
TFPB membrane	NPOE 400 uL; PVC 200 mg	TFPB 1.5 mg
NaISE		TFPB 1.5 mg; Na^+^ ionophore

## Data Availability

The data presented in this study are available on request.

## Abbreviations

The following abbreviations are used in this manuscript:

PADE	phosphoric acid di-n-decyl ester
TFPB	Bis[(12-crown-4)methyl] 2-dodecyl-2-methylmalonate
NaISE	Na^+^ ion-selective electrode
BCAAs	branched-chain amino acids
PVC	polyvinyl chloride
